# Supplementary material for: Ultra-sensitive metaproteomics redefines the dark metaproteome, uncovering host-microbiome interactions and drug targets in intestinal diseases
Source: Nat Commun. 2025 Jul 18;16:6644. doi: 10.1038/s41467-025-61977-7 (PMC12274446; doi:10.1038/s41467-025-61977-7)
Supplement: Supplementary file 1 — Supplementary Information [file 41467_2025_61977_MOESM1_ESM.pdf]

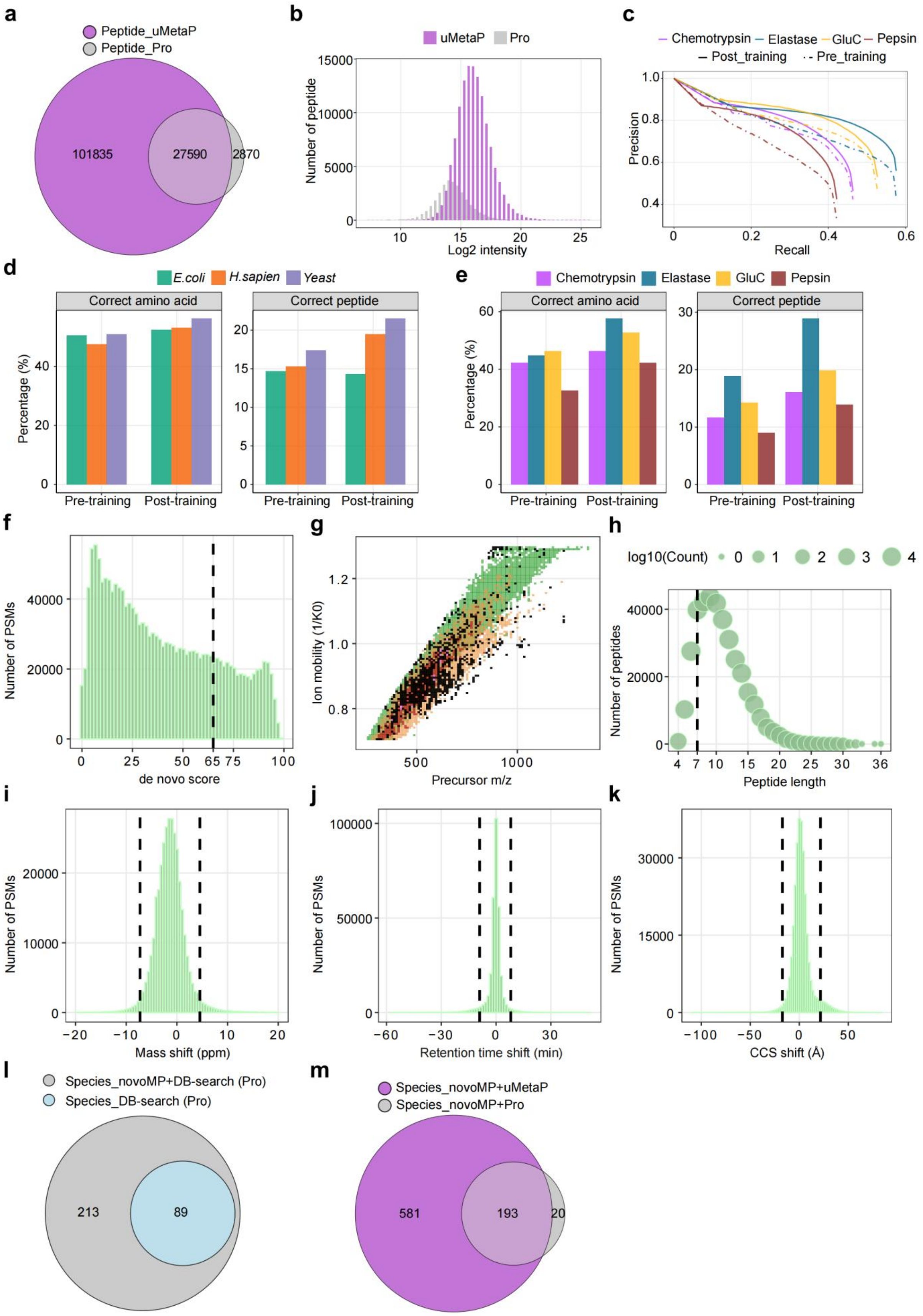

Supplementary Fig. 1: **Comprehensive evaluation of novoMP performance in peptide identification and taxonomic annotation. a** Overlap of peptides identified by current uMetaP (magenta) and previous timsTOF Pro workflows (gray) using same pre-fractionated

samples. **b** Log2 intensity distribution of peptides identified by uMetaP and previous timsTOF Pro workflows. **c** Precision-recall curves comparing pre-training and post-training performance of Novor algorithm across enzymes (Chemotrypsin, Elastase, GluC, and Pepsin). **d** Percentage of correct amino acid and peptide identifications for in a dataset generated from species-mix samples (*E. coli*, *H. sapiens*, and yeast) in pre- and post-training conditions of Novor. **e** Percentage of correct amino acid and peptide identifications across datasets prepared with various enzymes. **f-k** Filtering and validation metrics applied to novoMP-derived PSMs: **f** Distribution of *de novo* scores, with the dotted line indicating the filtering threshold (score value = 65) for high-confidence matches. **g** Distribution of precursor charge states of *de novo* sequenced PSMs. Black-dots represent singly charged precursors that were excluded for further processing. **h** Stats of peptide length and corresponding counts. The black-dotted line indicates the cut-off of 7 amino acids. **i** Mass shift distribution of *de novo* PSMs. The black-dotted lines indicate the upper (+4.54 ppm) and lower (-7.28 ppm) cut-off to ensure 95% of the data under the distribution. **j** Distribution of retention time shifts between observed and predicted values. The black-dotted lines indicate the upper (+8.04 min) and lower (-8.99 min) cut-off to retain 95% of the data. **k** Distribution of cross-collision section (CCS) differences between observed CCS and predicted CCS. The black-dotted lines indicate the upper (+21.59 Å) and lower (-17.25 Å) cut-off to keep 95% of the data under the distribution. **l** Venn diagram showing species identified by applying novoMP (gray) and classic DB-search strategy (light blue) in a dataset acquired using our previous timsTOF Pro workflow. **m** Annotated species comparison by applying novoMP to current uMetaP workflow (magenta) and previous timsTOF Pro workflows (gray) using same pre-fractionated samples. Source data for Supplementary Fig. 1c, 1d, 1g and 1e are provided in the Source Data file.

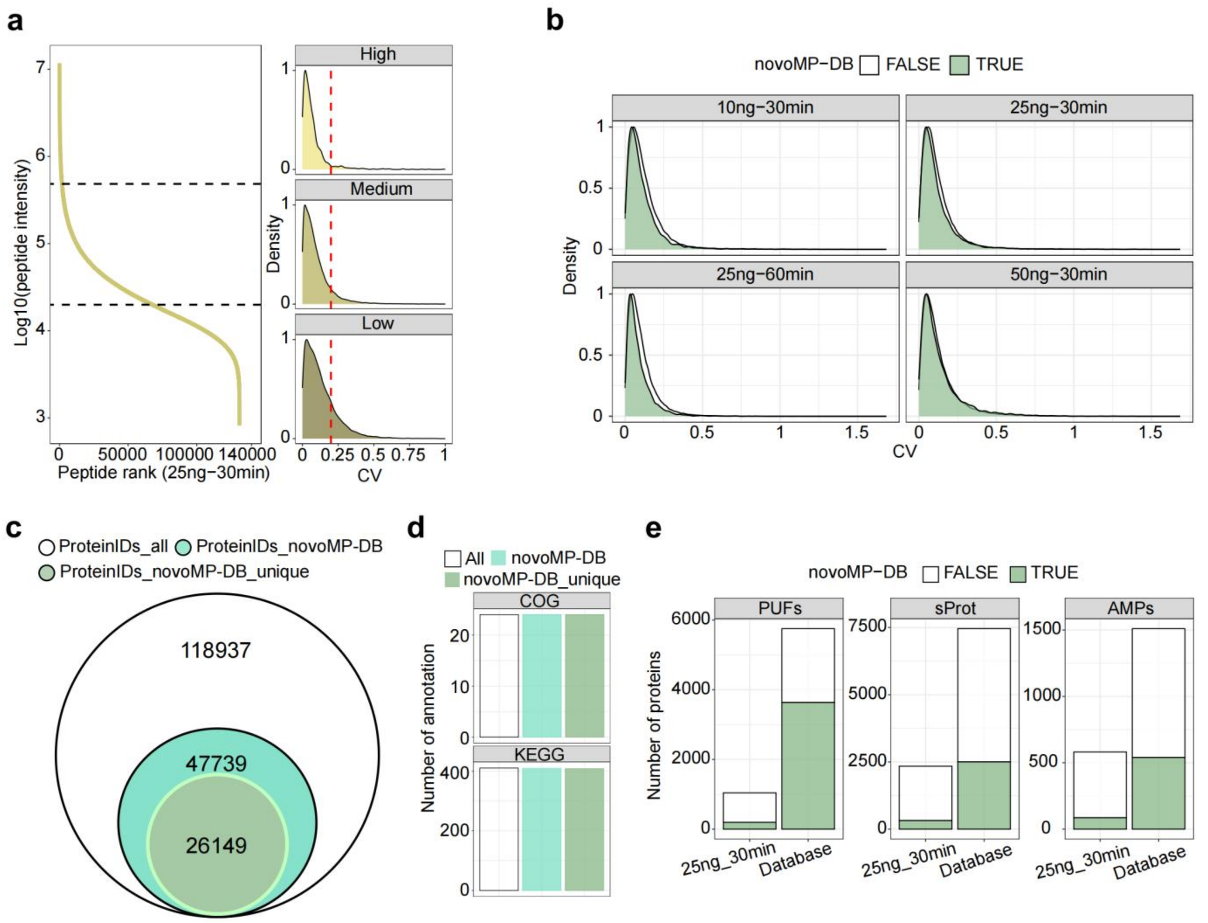

Supplementary Fig. 2: **Validation of quantitative precision and functional annotations enabled by uMetaP and novoMP in complex metaproteomic datasets. a** Peptide intensity distribution and quantitative precision analysis for 25 ng of peptides analyzed with a 30-minute LC gradient. Left: Log10 intensity distribution ranked by peptide abundance, categorized into high, medium, and low-intensity groups. Right: Density plots of the coefficient of variation (CV) for each intensity group, with the red dashed line indicating a CV threshold of 0.2. **b** Density plots of CV values (triplicates) across varying sample loadings (10 ng to 50 ng) and LC gradient lengths (30 to 60 minutes). Peptides identified by novoMP-DB (green) demonstrate comparable or superior quantitative precision to database-searched peptides across all conditions. **c** Overlap of all identified protein groups (ProteinIDs_all), those identified with novoMP-DB (ProteinIDs_novoMP-DB; i.e., at least one protein in the protein group was derived from novoMP-DB), and those uniquely identified by novoMP-DB (ProteinIDs_novoMP-DB_unique; i.e., all proteins in the group were from novoMP-DB). **d** Functional annotations (COG and KEGG) of all identified protein groups (All), those identified with novoMP-DB (ProteinIDs_novoMP-DB), and those uniquely identified by novoMP-DB (ProteinIDs_novoMP-DB_unique). **e** Amount of PUFs, sProt, and AMPs experimentally detected using 25 ng with a 30-min gradient and present in the constructed microbial protein database. Source data for Supplementary Fig. 2a and 2b are provided in the Source Data file.

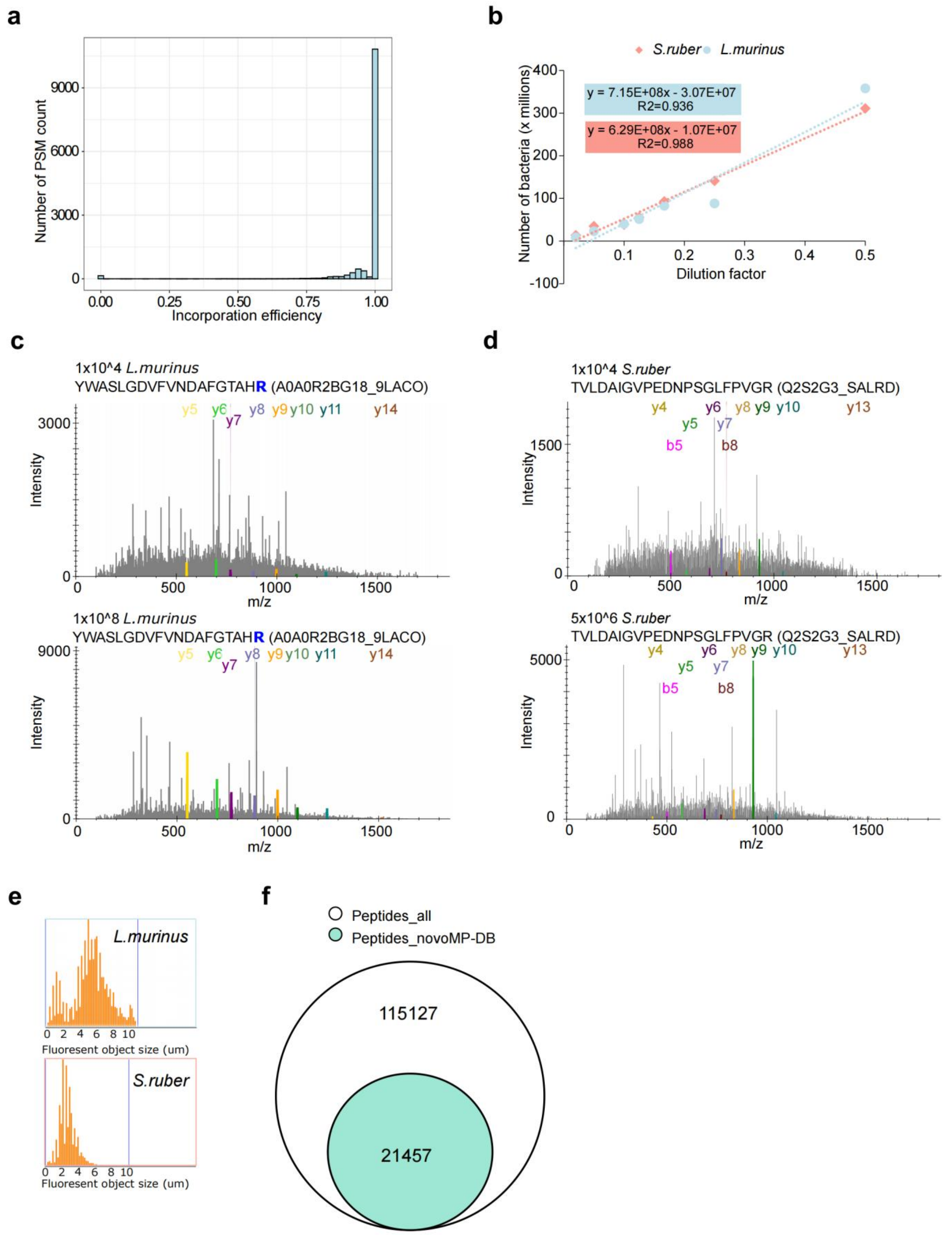

Supplementary Fig. 3: **Validation and characteristics of peptides and proteins identified in spiked-in bacterial experiments.** **a** Distribution of SILAC incorporation efficiency for *L. murinus*, showing an average incorporation efficiency of 97.42%. **b** Linear relationship between dilution factors and counted bactria for *S. ruber* and *L. murinus*. **c-d** Representative of identified MS/MS spectra of peptides from *L. murinus* (**c**) and *S. ruber* (**d**) at the LoD of 10^4 bacterial cells compared to same peptides at higher spike-in amounts. **e** Fluorescent

object size distribution for *L. murinus* and *S. ruber* measured during bacterial counting. **f** Venn diagram comparing the total peptides identified (Peptides_all) and those identified by novoMP-DB (Peptides_novoMP-DB).

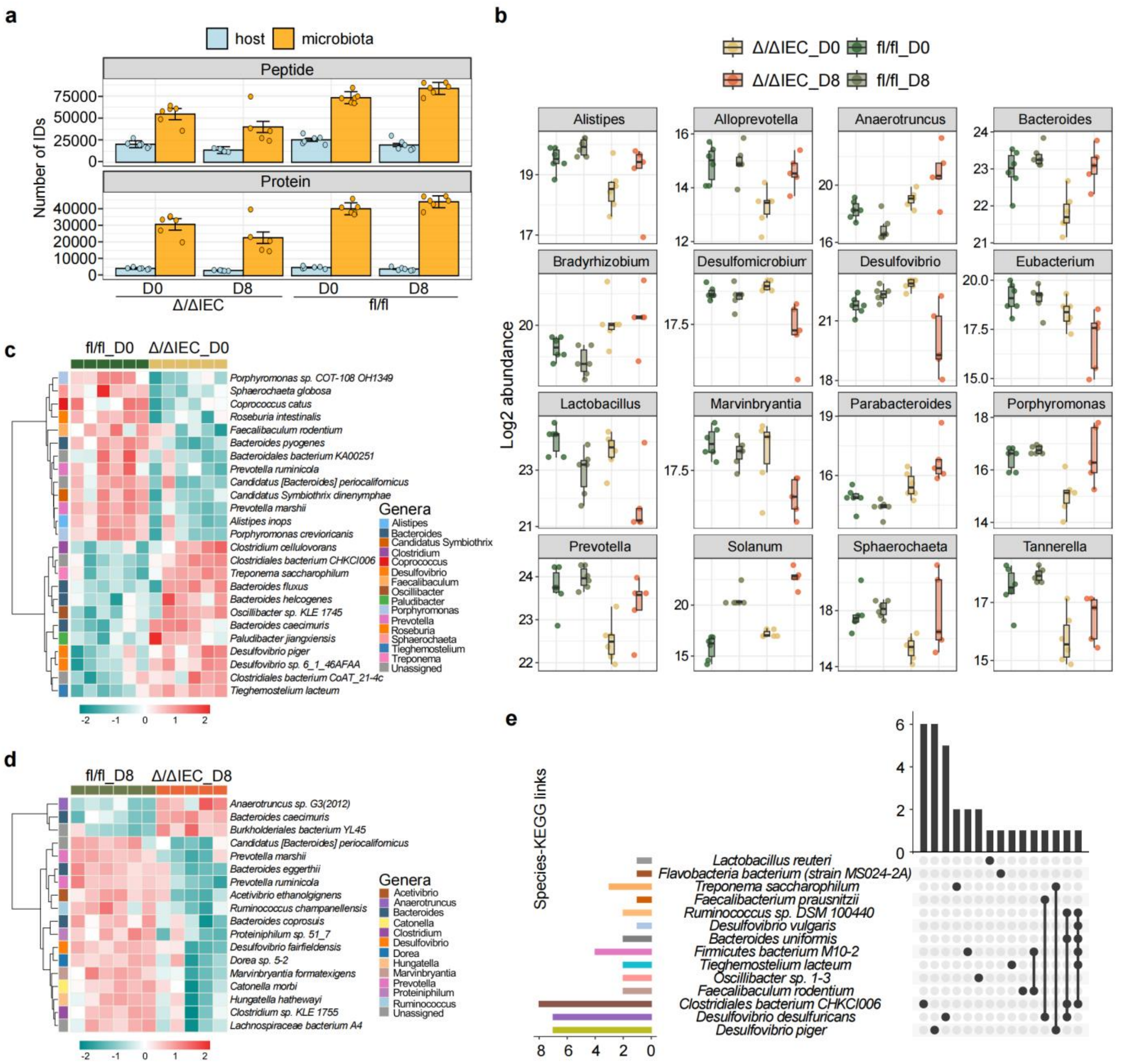

Supplementary Fig. 4: **Microbial taxonomic and functional changes during intestinal injury in response to mitochondrial dysfunction. a** Number of peptides and protein identifications for host and microbiota across D0 and D8 in control (Hsp60$^{fl/fl}$) and injured (Hsp60$^{\Delta/\Delta IEC}$) mice. Bar plots show the identifications with mean ± standard deviations. Sample sizes for experimental conditions are: Δ/ΔIEC_D0 = 6, fl/fl_D0 = 6, Δ/ΔIEC_D8 = 5, and fl/fl_D8 = 6. **b** Log2 abundance of 16 significantly altered genera in response to metabolic injury discovered by uMetaP. Box plots show the distribution of abundance per sample group. Each box represents the inter-quartile range, spanning from the 25th percentile (lower bound of the box) to the 75th percentile (upper bound). The center line indicates the median (50th percentile). **c-d** Abundances of differentially altered species at D0 (**c**) and D8 (**d**). The genera assignments of those species are colored and shown on the left of the heatmaps. Moderated two-sided t-tests was used followed by Benjamini-Hochberg method for p value adjustments. **e** UpSet plot showing uniqueness and shareness of significantly regulated KEGG pathways among 14 species at D0. Source data for Supplementary Fig. 4a, 4b, 4c and 4d are provided in the Source Data file.

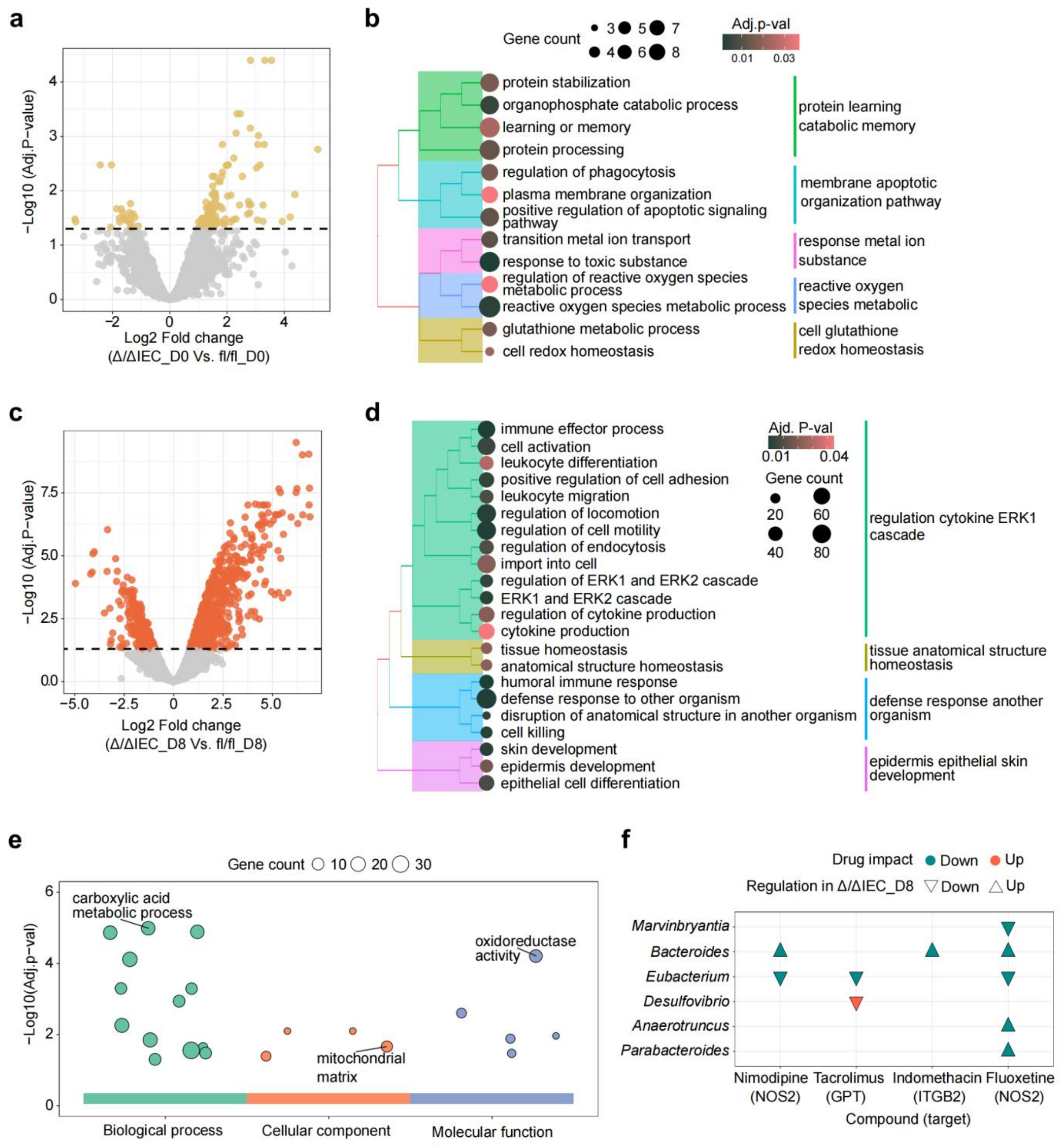

Supplementary Fig. 5: **Functional enrichment analysis of host proteome changes. a** Volcano plot showing log2 fold changes of significantly regulated proteins at D0 (Δ/ΔIEC_D0 vs. fl/fl_D0). Proteins with adjusted p-values ≤ 0.05 (moderated two-sided t-tests followed by Benjamini-Hochberg for adjusted p value) are highlighted. **b** Enriched biological processes from significantly regulated proteins at D0. **c** Volcano plot showing the log2 fold changes of significantly regulated proteins (Δ/ΔIEC_D8 vs. fl/fl_D8). **d** Functional enrichment analysis of significantly regulated proteins at D8 highlights pathways related to tissue homeostasis, epithelial development, cytokine regulation, and the pro-inflammatory ERK1/2 cascade. Gene counts and adjusted p-values are visualized by size and color, respectively. **e** GO enrichment of 33 proteins consistently regulated in mouse metaproteomics (colonic content), mouse targeted RNA analysis (colon tissues), and human transcriptomics datasets (Crohn's disease, ileum biopsy). The enriched terms are sized based on the number of genes

mapped. **f** Summary of drug effects on genera reported by Li L *et al.*[31] and the abundance changes in Δ/ΔIEC_D8 mice. Source data for Supplementary Fig. 5a and 5c are provided in the Source Data file.

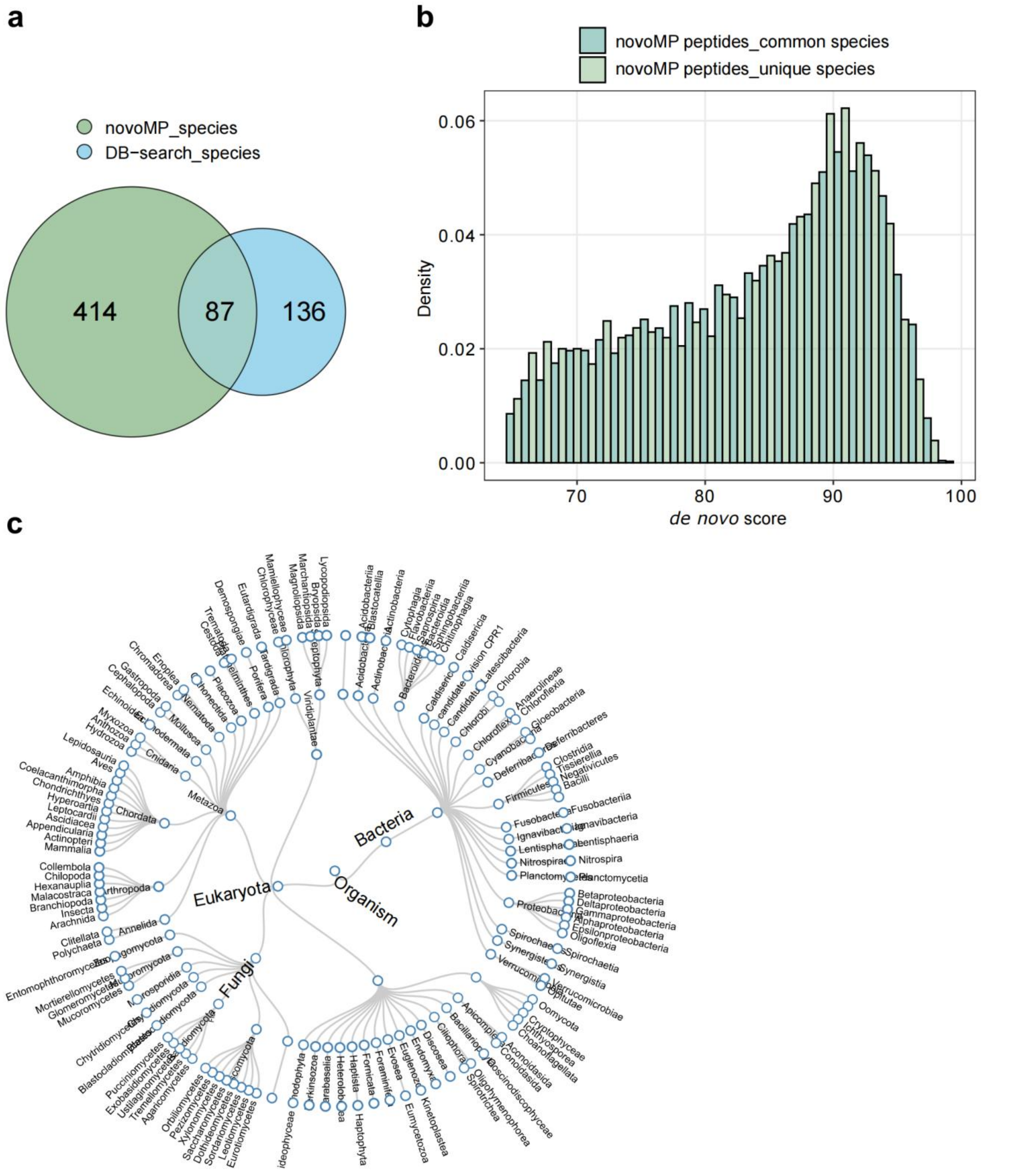

Supplementary Fig. 6: **Taxonomic coverage and confidence of species-level annotations from novoMP and DB-search workflows. a** Venn diagram showing the number of annotated species identified by peptides from the novoMP workflow (green) and classical database search (blue), applying a cut-off of at least three species-specific peptides. **b** Distribution of *de novo* scores for peptides assigned to the 87 species shared between both workflows and the 414 species uniquely identified by novoMP. **c** Circular phylogenetic tree illustrating the species identified exclusively through additional novoMP workflow. Source data for Supplementary Fig. 6b are provided in the Source Data file.

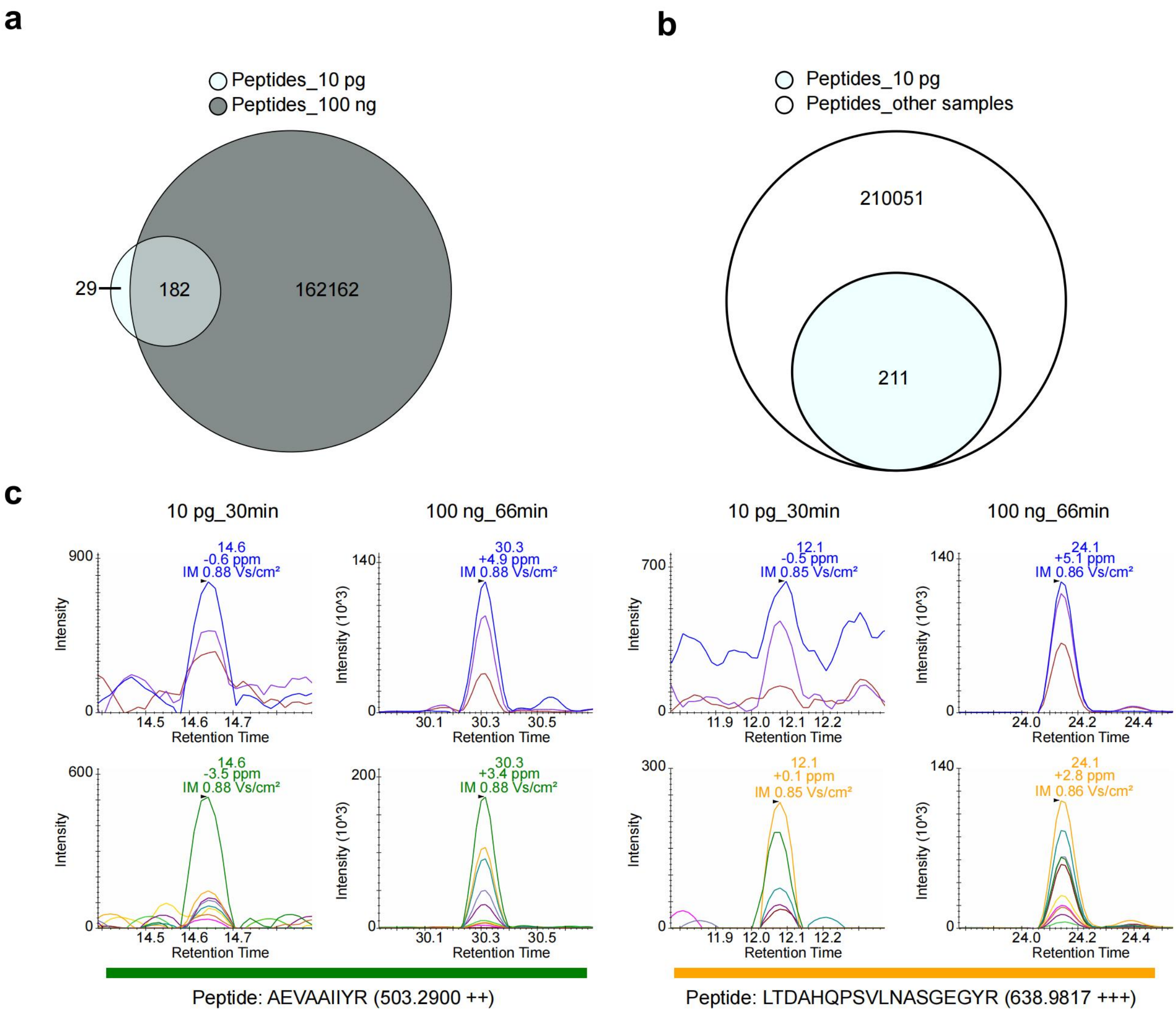

Supplementary Fig. 7: **Confidence assessment of peptides identified at low input levels. a** Venn diagram showing the overlap of peptide identifications between 10 pg and 100 ng input samples. Twenty-nine peptides were detected at 10 pg but not at 100 ng. **b** Venn diagram comparing peptides identified at 10 pg with all other higher-input samples. All 211 peptides identified at 10 pg were also observed in at least one other sample. **c** Extracted ion chromatograms for two representative peptides (from the 29 in Supplementary Fig. 7a) detected in 10 pg but not reported in 100 ng samples. Similar fragment ion patterns support confident identifications at 10 pg and suggest that absence in 100 ng is likely due to false negatives rather than false discovery in 10 pg.
