# Supplementary material for: Ultra-sensitive metaproteomics redefines the dark metaproteome, uncovering host-microbiome interactions and drug targets in intestinal diseases
Source: Nat Commun. 2025 Jul 18;16:6644. doi: 10.1038/s41467-025-61977-7 (PMC12274446; doi:10.1038/s41467-025-61977-7)
Supplement: Supplementary file 2 — Description of Additional Supplementary Files [file 41467_2025_61977_MOESM2_ESM.pdf]

Description of Additional Supplementary Files

File Name: Supplementary Data 1

Description: Taxonomy annotation of peptides detected from novoMP and classic DB-search.

File Name: Supplementary Data 2

Description: Identified microbial and host peptides and proteins using DIA-PASEF enabled by uMetaP.

File Name: Supplementary Data 3

Description: Identified PSMs of *L. murinus* for the calculation of labeling efficiency.

File Name: Supplementary Data 4

Description: Identified peptides of spike-in *L. murinus* and *S. ruber* at different bacterial numbers.

File Name: Supplementary Data 5

Description: Calculated biomass based on summed intensity of peptides annotated to corresponding taxa.

File Name: Supplementary Data 6

Description: Statistical comparisons of quantified species-KEGG pathways at D0 and D8.

File Name: Supplementary Data 7

Description: Statistical comparisons of quantified COG IDs between Δ/ΔIEC and fl/fl samples at D8.

File Name: Supplementary Data 8

Description: List of significantly enriched host pathways at D0 and D8.

File Name: Supplementary Data 9

Description: List of significantly regulated host proteins identified by uMetap that overlap with transcriptomic data from Crohn’s disease patients and mouse colonic samples.

File Name: Supplementary Data 10

Description: Enriched Gene Ontology terms of 33 proteins consistently regulated in mouse metaproteomics (colonic content), mouse targeted RNA analysis (colon tissues), and human transcriptomics datasets (Crohn’s disease, ileum biopsy).

File Name: Supplementary Data 11

Description: List of mapped drug-gene interactions.
